# Empirical Evidence for the Impact of Environmental Quality on Life Expectancy in African Countries

**DOI:** 10.5696/2156-9614-11.29.210312

**Published:** 2021-03-02

**Authors:** Sisay Demissew Beyene, Balázs Kotosz

**Affiliations:** 1 Faculty of Economics and Business Administration, Doctoral School of Economics, University of Szeged, Hungary; 2 College of Business and Economics, Department of Economics, Arsi University, Assela, Ethiopia; 3 IESEG School of Management, Paris, France

**Keywords:** environmental quality, environmental performance index, ecosystem vitality, life expectancy, Sustainable Development Goals, dynamic fixed effect, Africa

## Abstract

**Background.:**

Protecting the health of citizens is a central aim of sustainable development plans, due to the effect of health on social and economic development. However, studies show that environment-related diseases adversely affect the health status of a people, and this situation is worse for African countries. The Sustainable Development Goals (SDG) targets have included reducing environment-related deaths since 2015. However, there is a lack of empirical findings focused on the effects of environmental quality on life expectancy in Africa.

**Objectives.:**

The present study examined the impact of environmental quality on life expectancy in 24 African countries.

**Methods.:**

Time-series data ranging from 2000 to 2016 was used and the panel autoregressive distributed lag (ARDL)–dynamic fixed effect (DFE) model was employed to analyze the data.

**Results.:**

The results confirmed that, in the long run, improvements in environmental quality significantly increased life expectancy in the studied African countries during the study period. A unit increment in environmental performance index (EPI) and ecosystem vitality (EV) increased the life expectancy of Africans by 0.137 and 0.1417 years, respectively.

**Conclusions.:**

To the best of the authors' knowledge, this is the first empirical (econometric) study using a broad measurement (indicator) of environmental quality to investigate its impact on life expectancy in African countries. The study recommends that the introduction of environmentally friendly economies (like renewable energy, land, water, and waste management), legal, socio-economic, demographic, and technological measures are essential to reduce environmental pollution and improve life expectancy in Africa.

**Competing Interests.:**

The authors declare no competing financial interests.

## Introduction

According to the Alma-Ata Declaration, protecting human health is vital for human welfare and social and economic development.^1,2,3^ Healthy people can positively contribute to the economic growth and development of a country, directly and indirectly, by providing labor to different sectors. This improves per capita income and reduces poverty and income inequality. Governments should actively seek to preserve their people's lives and reduce the incidence of unnecessary mortality and avoidable illnesses.^4^

Multiple factors (economic, political, social, and environmental) affect human health. Globally, around one-quarter of all diseases and deaths (about 13 million deaths each year) are caused by environmental problems.^4–7^ Studies show that air pollution results in seven million deaths per year globally, more than 90% of the world's population breathes polluted air, and almost 3,000 million people still depend on polluting fuels.^7,8^ Studies also show that more than half of the world's population is living with unclean water, poor sanitation and hygiene, which causes more than 800,000 deaths each year.^7,8^ Furthermore, the lack of effective environmental management results in 400,000 deaths annually from malaria and 700,000 deaths from other vector-borne diseases.^7,9^ Statistics show that 23% of all deaths globally are environment-related. Specifically, there were 3.8 million environment-related deaths in Southeast Asia, 3.5 million in the Western Pacific, 1.4 million in Europe, 854,000 in the Eastern Mediterranean region, and 847,000 in the Americas. Moreover, according to the report of the World Health Organization (WHO),^10^ stroke, ischemic heart disease, accidental injury, cancer, chronic respiratory diseases, diarrheal disease, respiratory infection, neonatal conditions, malaria, and non-accidental injury are the ten most common causes of deaths due to environmental problems.

The story is quite similar when it comes to Africa; according to the WHO,^7^ the continent is fighting the world's biggest public health crisis. Around 2.2 million deaths each year are reported to be caused by environment-related diseases in Africa. Air pollution alone is responsible for 600,000 deaths every year across the continent. In Africa, approximately 66% of children live in homes where solid fuels are used for cooking and heating,^11^ which was responsible for 400,000 deaths in the region in 2017.^12^ Countries in sub-Saharan Africa (SSA) are also affected by serious environmental problems, such as soil erosion, deforestation, desertification, insect infestation, and wetland degradation.^13^ In addition, carbon dioxide (CO_2_) emissions are an important environmental problem in SSA countries, at over 0.8 metric tons per capita since 1990 and reaching a maximum of 0.93 metric tons in 2003.^14^ Rapid environmental degradation is another crucial problem in the Horn of Africa.^15^ According to Mutai,^16^ overgrazing, deforestation, shortage of water, loss of biodiversity, and industrial pollution in urban areas are the most serious environmental problems in East Africa and the Horn of Africa.

Policymakers regard the link between environmental quality and health status as a cause of increasing global concern and their 2030 agendas include plans to reduce risks to health from the environment. The global Sustainable Development Goals (SDGs) comprise seventeen goals in all. The third goal, titled ‘Good Health and Well-being', is concerned with issues that directly and indirectly concern the health status of humans.^10^ In particular, the third and sixth goals focus on the environment and health.^17^ Another SDG target is the reduction of the environmental impact of cities on human health by minimizing air, water, and soil pollution and putting into practice appropriate waste management.^18^ However, few empirical studies exist that assess the impact of environmental quality on life expectancy in African countries, even though these countries support people that are often disproportionately exposed to environment-related diseases. The few studies that do exist are often out of date. They therefore do not use the latest methodologies, and their measurements of environmental quality are too specific, i.e., carbon dioxide (CO_2_), sulfur dioxide (SO_2_), or other indicators. All of the above considerations lead to gaps in the literature, methodology, and appropriate measurements that this study aims to overcome. Specifically, this study addresses the methodology deficit by employing panel data autoregressive distributed lag (ARDL)–dynamic fixed effect (DFE) estimation, and the issue of measurement by using broad indicators (environmental performance index (EPI), and ecosystem vitality (EV)) to examine the impact of environmental quality on life expectancy in the case of 24 African countries from 2000 to 2016. Due to unavailability of data especially on target variables (environmental quality) this study is limited to only 24 African countries.

**Abbreviations**
*ARDL*: Autoregressive distributed lag
*DFE*: Dynamic fixed effect
*EPI*: Environmental performance index
*EV*: Ecosystem vitality
*OLS*: Ordinary least square
*SSA*: Sub-Saharan Africa

### Empirical literature review

This section presents empirical studies on the impact of the environment on human health in general even though most studies used proxy variables (life expectancy, mortality rate, and health expenditure) to measure health status. Table 1 presents information on author(s), year of publication, model adopted, scope of study in terms of time and number of case studies, and findings across studies. Table 1 also illustrates the range of different indicators, such as CO_2_, SO_2_, greenhouse gas emissions, and carbon monoxide, which are used to measure environmental quality.

**Table 1:** Empirical Literature

**Author(s)**	**Model type**	**Scope and case study**	**Summarized results**

*Mutizwa and Makochekanwa^2^*	Random effects and fixed effects	2000 and 2008, 12 SADC countries	Environmental factors account for about 38% of mortality in the SADC region

*Narayan and Narayan^19^*	Panel OLS and panel dynamic OLS estimators	1980 to 1999, Eight OECD countries	Carbon monoxide and sulfur oxide emissions have a positive effect on health expenditures
*Alvarez et al.^20^*	Pearson/Spearman correlation analysis	1997 to 2006, SSA	Education, sanitation, and economic factors contribute to reduce maternal mortality
*Mariani et al.^21^*	Overlapping generations model	132 countries	Life expectancy (longevity) and environmental quality positively correlated
*Sanglimsuwan^22^*	Panel fixed effects	1990, 1995, 2000, and 2005, for 80 countries	Access to quality water, improved sanitary facilities and population density are statistically significant variables affecting infant mortality rate
*Amjad and Khalil^23^*	ARDL	1970 to 2012, Sultanate of Oman	CO_2_ emissions negatively and significantly associated with life expectancy in the short-run, but positive and insignificant in the long run
*Yazdi et al.^24^*	ARDL	1967 to 2010, Iran	Income and pollutants are associated with health expenditures in both short run and long run
*Issaoui et al.^25^*	Fully modified OLS and dynamic OLS	1999 to 2010, MENA countries	CO_2_ emissions negatively affect life expectancy
*Fischer et al.^26^*	Cox proportional hazard models	2004 to 2011, Netherlands	Long-term exposure to to PM_10_ and NO_2_ was associated with non-accidental and cause-specific mortality for age ≥ 30 years
*Balan^27^*	Dumitrescu and Hurlin (2012) non-causality test	1995 to 2013, 25 EU member states	Causal relationship between environmental quality and health (life expectancy)
*Matthew et al.^28^*	ARDL	1985 to 2016, Nigeria	Greenhouse gas emissions reduce life expectancy, while government health care expenditure increases life expectancy
*Schwartz et al.^29^*	Causal modelling techniques	2000 to 2013, USA	Reducing PM_2.5_ concentrations increases life expectancy
*Nkalu and Edeme^30^*	GARCH	1960 to 2017, Nigeria	Environmental hazards proxied by CO_2_ emission from solid fuel consumption reduces life expectancy

Abbreviations: ARDL, Autoregressive distributed lag; EU, European Union; GARCH, Generalized auto regressive conditional heteroskedasticity; MENA, Middle East and North African countries; OECD, Organization for Economic Co-operation and Development; OLS, Ordinary least square; SADC, Southern African Development Community; SSA, Sub-Saharan Africa

Matthew *et al.*^28^ and Nkalu and Edeme^30^ have examined the impact of environmental pollution on life expectancy in Nigeria. However, findings concerning a single country (especially a country with an oil-based economy) cannot represent the majority of African countries and its policy recommendations may not, therefore, work for most African countries. Furthermore, these studies did not use a comprehensive measure of environmental pollution.

Studies by Alvarez *et al*.^20^ and Mutizwa and Makochekanwa^2^ used panel data models in the context of African countries. However, both of these studies are outdated, employed conventional methodologies, and did not use comprehensive measures of environmental quality, leading to methodological and measurement gaps. For example, Alvarez *et al*.^20^ employed Pearson correlation analysis, but this method only measures the strength and direction of a liner relationship between two variables. In panel data econometrics, this method is mostly used to summarize data, as an input into a more advanced analysis. Therefore, a Pearson correlation, which ignores the interactions of all other explanatory variables and focuses on the relationship between two variables only, is not sufficient to provide conclusions and set appropriate policy recommendations. Similarly, Mutizwa and Makochekanwa^2^ used a method which estimated fixed effects and random effects, which does not capture the dynamic nature of the countries and data. In addition, this study did not consider cross-sectional dependence among countries and their regressors. However, Pesaran^31^ noted that overlooking cross-sectional dependence, especially if it existed, leads to spurious results.

Therefore, the present study overcomes the limitation of previous empirical studies by focusing on Africa, employing a dynamic model along with cross-sectional dependence in its methodology, using a comprehensive measure of environmental quality, and examining the latest time period data available for 24 African countries (Algeria, Angola, Benin, Botswana, Cameroon, Congo, Cote d'Ivoire, Democratic Republic of the Congo, Egypt, Ethiopia, Gabon, Ghana, Kenya, Morocco, Mozambique, Namibia, Nigeria, Senegal, South Africa, Sudan, Tanzania, Togo, Tunisia, and Zambia).

## Methods

Depending on the nature of the data, panel time series models are classified as static models (pooled OLS, fixed effects (FE), and random affects (RE)) or dynamic models (generalized methods of momentum (GMM) and ARDL). Unlike static models, dynamic models capture the dynamic nature of the data. The ARDL model differs from other dynamic models in that it can be used if the variables in the model have the same, different, or mixed order of integration. Furthermore, it provides both long-run and short-run estimation results at the same time^32,33^ and, even if endogeneity problems exist, ARDL models provide reliable coefficients.^34^ The DFE estimator is one of the methods used for estimation of panel ARDL. The DFE estimator imposes homogeneity restrictions on both the long-run and short-run estimation coefficients, although it allows the intercept to be heterogeneous.^35–37^ According to Salahuddin *et al.*^35^ the DFE model is efficient when countries have similar macroeconomic structures and temporary shocks (because the DFE captures heterogeneity by country-specific intercepts).

With the exception of a few countries, the economy of most of Africa can be characterized as low- and middle-income countries (LMICs), being predominantly agricultural with largely subsistence farming. In addition, since the launch of the millennium development goals in 2000 and the SDGs in 2015, the environmental and health policies of Africa are the same across countries. Furthermore, Africans have experienced temporary shocks arising from local laws, regulations, and political regimes. Therefore, based on the above evidence, employing the DFE approach of Pesaran *et al.*^38^ in this study is justified.

### Data type, sources, and data analysis

Since some data are lacking, especially on EPI and EV, the present study is focused on only 24 African countries from the year 2000 to 2016. In addition, missing values of environmental quality variables for 2011, 2013, 2015 were interpolated. Except for environmental quality, political stability, and mean schooling, all the data were collected from the World Bank *(Table 2).*

**Table 2:** Study Variables, Measurement, Data Sources, and Sampled Countries

***Variable***	***Definition and measurement***	***Data sources and links***

*LIFEXP*	Life expectance measured as life expectancy at birth, total (years)	WB database^39^ https://datacataloe.worldbank.org/dataset/world-development-indicators
*EPI*	Environmental performance measured as an index from 0 to 100	YCELP^40^ https://sedac.ciesin.columbia.edu/data/set/epi-environmental-performance-index-2016
*EV*	Ecosystem vitality measured as an index from 0 to 100	YCELP^40^ https://sedac.ciesin.columbia.edu/data/set/epi-environmental-performance-index-2016
*GDPPC*	GDP per capita (current US$)	WB database^39^ https://datacataloe.worldbank.org/dataset/world-development-indicators
*POPDEN*	Population density measured as people per sq. km of land area	WB database^39^ https://datacataloe.worldbank.org/dataset/world-development-indicators
*URBAN*	Urban population (% of total population)	WB database^39^ https://datacataloe.worldbank.org/dataset/world-development-indicators
*POLITY 2*	Political stability indicator (from −10 to +10) measured as the country’s election competitiveness and openness, the nature of political involvement and the degree of checks on administrative authority	Polity IV database^41^ http://www.svstemicpeace.org/inscr/
*GOVEXP*	Domestic general government health expenditure (% of GDP)	WB database^39^ https://datacatalog.worldbank.org/dataset/world-
*UNEMPL*	Unemployment, total (% of the total labor force) (modeled ILO estimate)	WB database^39^ https://datacataloe.worldbank.org/dataset/world-development-indicators
*MNSCHOOL*	Mean years of schooling (years)	United Nations Development Programme (UNDP)^42^ database http://hdr.undp.ore/en/data

Abbreviations: GDP, Gross domestic product; ILO, International Labor Organization; WB, World Bank; YCELP, Yale Center for Environmental Law and Policy

Measuring environmental quality is a complicated task since it involves different indicators such as CO_2_ levels, access to safe water, and sanitation facilities. This study, however, used a broad and comprehensive indicator called the EPI, and its component EV. The EPI takes into account both environmental health (EH) and EV. Environmental health accounts for 30% of EPI and encompasses child mortality, indoor air pollution, levels of particulate matter, access to drinking water, and access to sanitation. However, according to the Yale Center for Environmental Law and Policy (YCELP) data, the EV contributes 70% to the EPI and contains seventeen indicators.^40^ The EPI is calculated as a weighted average of all indicators of EH and EV; each one converted to a proximity-to-target measure with a theoretical range of 0–100.^21^ Likewise, EH and EV are computed as a weighted average of all five and 17 indicators, respectively. Therefore, EPI, EH, and EV can all take values ranging between (0–100).

Due to the non-stationary behavior of EH, this study used only EPI and EV as proxy variables for environmental quality. First, using the EPI is essential for avoiding a biased view of environmental quality.^21^ Likewise, using EV is vital to confirm the estimation result of a model using EPI because 70% of EPI is derived from EV. This approach makes this study different from others because it measures the broad concepts of the environment.

The other target variable in this study is health status, which is a dynamic and broad assessment of the physical, social, spiritual, and mental well-being of the population that translates to quality of life and not merely the absence of illness.^2,43^ However, since it is too challenging to incorporate all the indicators of health status, this study used a proxy variable called life expectancy to measure health status.

### Model specification, estimation technique, procedures, ARDL model specification

The study adopted the Grossman^44^ model to examine the impact of environmental quality on the health status of 24 African countries (where life expectancy at birth is used as a proxy measure). This model is also used by studies conducted by Fayisa and Gutama^45^ and Mutizwa and Makochekanwa.^2^ However, unlike the log-linear model of Equation 1, most empirical studies used life expectancy without transforming to logarithm and hence, this study specified a linear model, which is expressed in Equation 1.


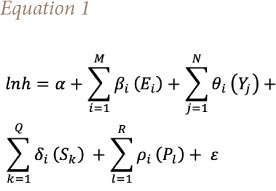


Initially, the Grossman^44^ model did not include political factors. However, this study extended the Grossman^44^ model by considering the impact of political stability on life expectancy.

Equation 1 describes the sum of variables that explain environmental (EPI and EV), economic (GDPPC and GOVEXP), social (POPDEN, URBUN, UNEMPL, MNSCHOOL), and political (POLITY2) factors, where *lnh* is the natural logarithm of a health status measured by life expectancy at birth, *E_i_* is a vector of environmental factors, *Y_j_* is a vector of economic variables, *S_k_* is a vector of social variables, *P_l_* is a vector of political variables, and *E_i_, Y, S_k_*, and *P_l_* are expressed as either index, percentage, or number of years (where =1,2…*M*; *j*=1,2…*N*; *k*=1,2…*Q*; l=1,2…*R*). *β_i_, θ_i_, δ_i_,* and *ρ_i_* are vector of coefficients while *α* is an estimate of the initial health stock of the region and *ε* is the error (disturbance) term. All variables are assumed to be measured in the same time period.

The present study adopted the Grossman,^44^ Fayisa and Gutama,^45^ and Mutizwa and Makochekanwa^2^ models. However, it modified (the dependent variable in this study is without logarithm) and extended the adopted models and formulated Equation 2.

In Equation 2, the expected hypothesized signs of explanatory variables are set in parentheses. This study used the panel ARDL estimation in order to achieve its objective. In addition, it constructed and estimated two models to investigate the independent impacts of EPI and EV on life expectancy. The general panel ARDL models of this study are specified in Equations 3 and 4.


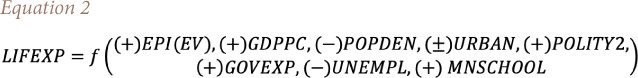



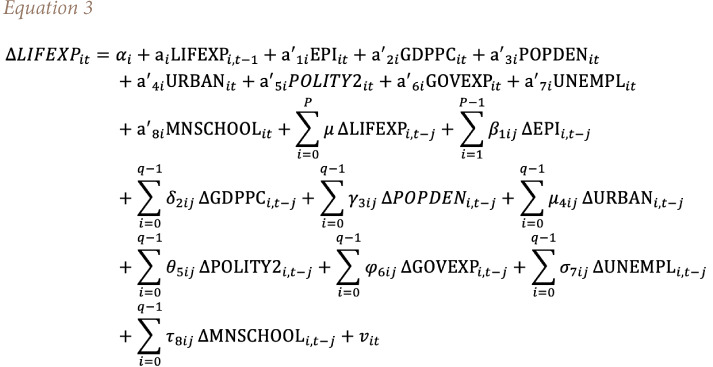



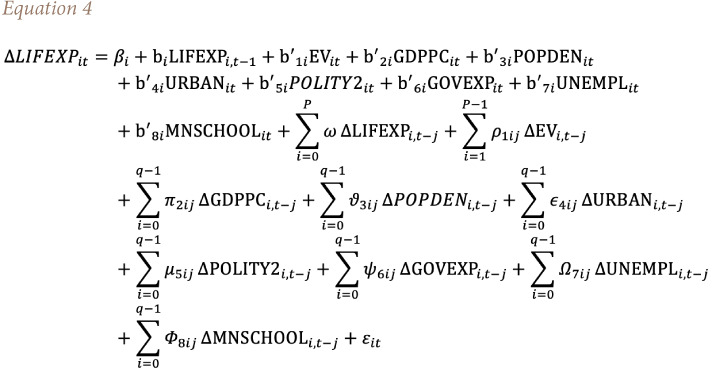


## Results

Table 3 provides descriptive and econometric results which are inputs and preconditions for more advanced analysis. Specifically, it has information about descriptive statistics, cross-sectional dependence, unit root, and co-integration tests of the models.

**Table 3:** Descriptive Statistics Across Study Variables, Cross-Sectional Dependence, Unit Root, and Cointegration Tests

*Variables*	**Obs**	**Mean**	**SD**	**Min**	**Max**

*LIFEXP*	408	59.348	7.652	44	76.298
*EPI*	408	48.076	7.15	24.64	77.28
*EV*	408	53.535	10.649	16.84	74.09
*GDPPC*	408	2076.198	2128.516	111.927	10809.65
*POPDEN*	408	51.585	40.65	2.179	204.17
*URBUN*	408	46.482	15.62	14.7	88.55
*POLITY2*	408	2.267	4.8778	−7	9
*GOVEXP*	408	1.873	1.301	0.062	6.762
*UNEMPL*	408	10.424	8.584	0.69	47.96
*MNSCHOOL*	408	5.296	8.584	1.5	10.2

***Cross-sectional dependence***		

***Tests***	Model 1	Model 2

*Pesaran’s test of cross-sectional independence*	0.653	0.819
*The average absolute value of the off-diagonal elements*	0.515	0.513
*Probability*	0.5139*	00..4126*

***Panel unit root test***			

*Variables*	**Statistics**	**Values**	**Order of integration**

*LIFEXP*	LLC	−17.613***	I(0)
	IPS	−23.105***	I(0)
	ADF	247.289***	I(0)
*EPI*	LLC	−5.017***	I(1)
	IPS	−8.831***	I(1)
	ADF	184.26***	I(1)
*EV*	LLC	−7.500***	I(1)
	IPS	−9.692***	I(1)
	ADF	201.65***	I(1)
*GDPPC*	LLC	−12.841***	I(1)
	IPS	−9.32678***	I(1)
	ADF	166.866***	I(1)
*POPDEN*	LLC	−9.769***	I(1)
	IPS	−7.396***	I(1)
	ADF	81.175***	I(1)
*URBUN*	LLC	−28.227***	I(1)
	IPS	−19.453***	I(1)
	ADF	95.408***	I(1)
*POLITY2*	LLC	−10.151***	I(1)
	IPS	−6.703***	I(1)
	ADF	91.772***	I(1)
*GOVEXP*	LLC	−11.721***	I(1)
	IPS	−10.948***	I(1)
	ADF	189.808***	I(1)
*UNEMPL*	LLC	−28.408***	I(1)
	IPS	−11.483***	I(1)
	ADF	132.527***	I(1)
*MNSCHOOL*	LLC	−12.040***	I(1)
	IPS	−11.996***	I(1)
	ADF	209.019***	I(1)

***Kao Cointegration test***				

	**Model 1**		**Model 2**	

	**t-Statistic**	**Prob.**	**t-Statistic**	**Prob.**

***ADF***	−2.746	0.0030***	−2.725	0.0032^***^
***Augmented Dickey-Fuller Test Equation***	0.156		0.156	
***HAC variance***	0.378		0.378	

Abbreviations: Obs- number of observations; SD- standard deviation; Min- minimum; Max- maximum; prob- probability; HAC- heteroskedasticity and autocorrelation constant; LLC- Levin, Lin and Chu; IPS- Im, Pesaran and Shin; ADF- augmented Dickey-Fuller test.

^*^,^***^⇒ no cross-sectional dependence and significance at 1% level, respectively.

Source: Computed by the authors - both unit root and cointegration tests computed using Eviews 10. However, the descriptive statistics and cross-sectional dependency test were computed using Stata 15. Variable definitions can be found in Table 2.

Following the basic diagnostic and panel data econometric tests which are presented above, Table 4 presents the long-run and short-run estimation results.

**Table 4:** Estimated Long-run and Short-run Coefficients using the Dynamic Fixed Effects Approach

	**Model 1**			**Model 2**		

**Long-run results**						

Variables	Coeff	SE	Prob	Coeff	SE	Prob

EPI	0.137	0.0692	0.048**	—	—	—
EV	—	—	—	0.1417	0.0545	0.009***
GDPPC	0.0055	0.0012	0.000***	0.0056	0.0012	0.000***
POPDEN	−0.3085	0.1430	0.031**	−0.2732	0.1446	0.059*
URBUN	1.0925	0.2167	0.000***	1.1534	0.2226	0.000***
POLITY2	−0.0789	0.1828	0.666	−0.0938	0.1872	0.616
GOVEXP	−2.119	0.6506	0.001***	−2.3063	0.6835	0.001***
UNEMPL	0.5422	0.1766	0.002***	0.5452	0.1812	0.003***
MNSCHOOL	−1.3177	1.1524	0.253	−1.0843	1.1609	0.350

**Short-run results**						

ECM	−0.0423	0.008	0.000***	−0.0410	0.0080	0.000***
D(EPI)	−0.0074	0.0037	0.050**	—	—	
D(EV)	—	—	—	−0.0052	0.0023	0.025**
D(GDPPC)	−0.00011	0.00003	0.001***	−0.00011	0.000031	0.000***
D(POPDEN)	0.3179	0.1941	0.101	0.2695	0.1937	0.164
D(URBUN)	0.8691	0.1024	0.000***	0.8583	0.1013	0.000***
D(POLITY2)	0.0020	0.0107	0.847	0.0022	0.0107	0.833
D(GOVEXP)	0.0555	0.0334	0.096*	0.0587	0.0331	0.077*
D(UNEMPL)	−0.0647	0.0130	0.000***	−0.0639	0.0129	0.000***
D(MNSCHOOL)	1.1326	0.0821	0.106	−0.1246	0.0816	0.127
CONSTANT	0.1912	0.3671	0.602	−0.0403	0.3766	0.915

Abbreviations: Coeff, Coefficient; Prob, Probability; SE, Standard error

^*^ Significant at 10% level,

^**^ significant at 5% level,

^***^significant at 1% level Variable definitions can be found in Table 2.

In addition to the results in Table 4, a causality test was conducted to check the robustness of the results *(Table 5).*

**Table 5:** Dumitrescu and Hurlin Panel Causality Tests

**Null hypothesis**	**W-Stat.**	**Zbar-Stat.**	**Prob**
**EPI ↛ LIFEXP**	13.3158	16.4172	0.0000***
**EV ↛ LIFEXP**	13.7775	17.1180	0.0000***
**GDPPC ↛ LIFEXP**	4.70434	3.34596	0.0008***
**POPDEN ↛ LIFEXP**	221.369	332.220	0.0000***
**URBUN ↛ LIFEXP**	112.342	166.729	0.0000***
**POLITY2 ↛ LIFEXP**	NSM	NSM	NSM
**GOVEXP ↛ LIFEXP**	10.0974	11.5320	0.0000***
**UNEMPL ↛ LIFEXP**	27.1988	37.4902	0.0000***
**MNSCHOOL ↛ LIFEXP**	22.8536	30.8946	0.0000***

Note: ^***^, significant at 1% level and implies we reject the null hypothesis that the independent variable does not homogeneously cause LIFEXP (see Eviews 10^46^ user guide pp. 1012).

NSM: near singular matrix. Variable definitions can be found in Table 2.

Source: Computed using EViews 10

Even though the present study estimated linear models *(Equation 2)*, it also estimated the adopted log-linear model of Grossman,^44^ Fayisa and Gutama,^45^ and Mutizwa and Makochekanwa^2^ to check the robustness (sensitivity analysis) of the results in Table 6 and 7.

**Table 6:** Robustness Check Estimation using the Dynamic Fixed Effects Approach

**Dependent Variable (LIFEXP)**				

	**Model 1**		**Model 2**	

**Long-run results**				

**Variables**	**Coeff**	**Prob**	**Coeff**	**Prob**
EPI	0.1134	0.077*	—	—
EV	—	—	0.1370	0.007**
GDPPCG^1^	0.2004	0.099*	0.2123	0.087*
POPDEN	−0.4019	0.005***	−0.3718	0.009***
URBUN	2.0818	0.000***	2.130	0.000***
P0LITY2	−0.0653	0.708	−0.0829	0.640
GOVEXP	−2.0002	0.001***	−2.2232	0.001***
UNEMPL	0.2142	0.088*	0.2027	0.109
MNSCHOOL	1.9987	0.036**	2.2310	0.024**

**Short-run results**				

ECM	−0.0513	0.000***	−0.0501	0.000***
D(EPI)	−0.0150	0.000***	—	
D(EV)	—	—	−0.0102	0.000***
D(GDPPCG)	−0.0031	0.499	−0.0035	0.447
D(POPDEN)	0.2781	0.216	0.2337	0.298
D(URBUN)	0.6321	0.000***	0.6290	0.000***
D(P0LITY2)	0.0108	0.380	0.0110	0.372
D(GOVEXP)	0.0841	0.031**	0.0893	0.021**
D(UNEMPL)	−0.0604	0.000***	−0.0584	0.000***
D(MNSCHOOL)	−0.0283	0.761	−0.0371	0.689
CONSTANT	−1.7567	0.000***	−1.988	0.000***

Abbreviations: Coeff, Coefficient; Prob, Probability

^*^ Significant at 10 % level,

^**^ significant at 5% level,

^***^ significant at 1 % level

Source: Computed by the authors using Stata 15

^1^ GDP per capita growth (annual %) – obtained from WB database^39^

**Table 7:** Robustness Check Estimation using the Dynamic Fixed Effects Approach

**Dependent variable (LNLIFEXP^2^)**				

	**Model 1**		**Model 2**	

**Long-run results**				
**Variables**	**Coeff**	**Prob**	**Coeff**	**Prob**

EPI	0.0018	0.057*	—	—
EV	—	—	0.0021	0.004**
GDPGR^3^	0.0037	0.051*	0.0039	0.041**
POPDEN	−0.0058	0.005***	−0.0053	0.011**
URBUN	0.0311	0.000***	0.0317	0.000***
POLITY2	−0.0008	0.747	−0.0011	0.679
GOVEXP	−0.0313	0.001***	−0.0346	0.000***
UNEMPL	0.0032	0.090*	0.0030	0.114
MNSCHOOL	0.0271	0.062*	0.0307	0.039**

**Short-run results**				

ECM	−0.0605	0.000***	−0.0593	0.000***
D(EPI)	−0.0002	0.000***	—	
D(EV)	—	—	−0.00018	0.000***
D(GDPGR)	−0.00008	0.356	−0.00009	0.303
D(POPDEN)	0.0048	0.228	0.00401	0.322
D(URBUN)	0.0115	0.000***	0.0114	0.000***
D(POLITY2)	0.00018	0.422	0.00018	0.412
D(GOVEXP)	0.00145	0.040**	0.0015	0.028**
D(UNEMPL)	−0.0010	0.000***	−0.00105	0.000***
D(MNSCHOOL)	−0.00023	0.891	−0.00039	0.813
CONSTANT	0.1611	0.000***	0.153	0.000***

Abbreviations: Coeff, Coefficient; Prob, Probability

^*^ Significant at 10% level,

^**^ significant at 5% level,

^***^significant at 1% level Variable definitions can be found in Table 2.

Source: Computed by the authors using Stata 15

^2^ Natural logarithm of life expectance measured as life expectancy at birth, total (years) – obtained from WB database^39^

^3^ GDP growth rate (annual %) – obtained from WB database^39^

## Discussion

Table 3 shows that the mean of the dependent variable was 59.348 and the range was between 44 and 76.298 years, indicating low variation. Similarly, EPI and EV, which are the target-independent variables, had means of 48 and 53.5, respectively, with a range between 24.64 and 77.28 for EPI and between 16.84 and 74.09 for EV, indicating low variation. Generally, for all the variables, except for population density and GDPPC, the range of variation was not very high. Table 3 presents more information about other variables in the model.

Panel-data models are likely to have cross-sectional dependence in the errors. Hence, the present study (uniquely) tested cross-sectional dependence using Pesaran's test.^47^ The results confirm that, in both models, there is no cross-sectional dependence among African countries included in the study *(Table 3).* Following the cross-sectional dependency test, the study examined the stationarity (unit root) of variables in the models. Based on the result of cross-sectional dependence, unit root tests are classified into two groups – the first generation and second-generation unit root tests. The first-generation panel stationarity test^48–50^ can be used when there is no cross-sectional dependence, but the second-generation panel unit root test, Pesaran's test,^31^ is used when there is cross-sectional dependence. Therefore, since there is no cross-sectional dependence in either of our models, this study used first generation panel unit root tests *(Table 3).* The results show that all variables, except LIFEXP, are I(1). Since all variables in the model are I(0) and I(1), employing panel ARDL estimation is justified.

Furthermore, the panel cointegration test is essential to understand the long-run relationship among the variables in the model. The most common panel cointegration tests when there is cross-sectional independence are the Pedroni test,^51,52^ the Kao residual cointegration test, and the Fisher-type cointegration test. To clearly understand the characteristics of the three types of cointegration tests, refer to Beyene and Kotosz.^14^ The present study used the Kao residual cointegration test due to the large number of variables in the model *(Table 3).* The results confirm the existence of a long-run relationship among the variables in both models, which leads the study for long-run and short-run estimations.

### Long-run and short-run results

The present study estimated two different models to obtain robust and reliable results concerning the impact of environmental quality on life expectancy. Both models showed that improvements in environmental performance and ecosystem viability positively and significantly increased life expectancy. A unit increment in EPI and EV increases life expectancy in Africa by 0.137 and 0.1417 years, respectively *(Table 4).* When we compare these results with other variables, their influence seems small, but this might be due to differences in measurement. That means that all independent variables are measured in either percentage or monetary terms; however, EPI, EV, and POLITY 2 are indices and the dependent variable (life expectancy) is measured in number of years. Therefore, these measurement differences can make the effect of EPI and EV on life expectancy smaller than those variables measured as a percentage.

The estimated results can be explained by the fact that improvements in environmental quality can reduce pollution (air, noise, chemicals, water) and the loss of natural areas, which also contribute to a substantial reduction in rates of environment-related diseases. This study suggested one resource which is available in Africa to improve environmental quality and life expectancy. The most important renewable energy, which also greatly contributes to reducing greenhouse gas emissions and global warming, is hydropower.^53^ Relative to other continents, Africa is the least effective at producing and exploiting the potential of hydropower. Studies suggest that Africa holds about 12% of global hydropower potential and a technically feasible output of 1800 TWh/year.^54^ However, currently Africa produces only 3% of the world's hydropower and exploits less than 10% of its potential.^54^ This suggests that if Africa utilized its hydropower potential effectively it could be expected to simultaneously reduce environmental pollution and improve life expectancy. Furthermore, the results of this study agree with those of other studies,^21–23,25,2,27–30^ even though they used different indicators to measure environmental quality. Hence, the present study confirms that environmental quality is crucial to increasing life expectancy in African nations.

There are several reasons why the results of the present study are in agreement with the findings of other studies.^21–23,25,2,27–30^ For example, the results are similar to those of similar to a study by Mariani *et al.*^21^ which used life expectancy at birth (in years) and EPI to measure the target variables which may have produced similar results. Similarly, to measure health status, this study and others^22,23,25,2,27–30^ used a better and more easily available proxy (either life expectancy or mortality rate), which may explain the similarities in the findings. The data for the dependent variable of this study and most other studies^22,23,2,28,30^ were sourced from the World Bank. Furthermore, a few other studies^23,28^ employed the latest methodology (ARDL). Therefore, the finding of this study, that environmental quality significantly increases the life expectancy of people in African countries, is similar to the conclusion of other studies, even though their explanations may be different. This means they found that environmental quality/pollution proxies using various indicators can increase/decrease life expectancy or reduce/increase mortality rate.

Similarly, per capita GDP and urbanization significantly increase life expectancy in African countries. In Model 1, a unit increment in per capita GDP or urbanization improves the life expectancy of Africans by 0.0055 or 1.0925 years, respectively. Likewise, in Model 2, a unit increment in per capita GDP or urbanization improved life expectancy by 0.0056 or 1.1534 years, respectively *(Table 4).* The positive impact of per capita GDP on life expectancy agrees with the relationship described by the “Preston curve”, which implies that, on average, individuals born in wealthier countries can expect to live longer than those born in developing countries.^55^ In other words, when the economy improves, the per capita income of individuals increases and hence they have better health.

The present study also shows unemployment significantly increases life expectancy, which is an unexpected but not surprising result. This finding may be due to the inappropriateness of the global definition and measurement of unemployment in developing countries like those in Africa. Even though “unemployed” is generally defined as applying to a person who is actively searching for employment but unable to find work, due to the lack of well-structured and more formalized labor markets, most “unemployed” people in Africa are participating in the informal economy, which hides the adverse effects of unemployment. According to the International Labor Organization (ILO),^56^ in Africa, 85.8% of employment is informal, which suggests that unemployed people in Africa can gain income by participating in the informal economy and hence they can improve their health status.

Finally, population density significantly reduces life expectancy in African countries. For example, World Bank^57^ data shows there were 31.32 people per sq. km of land in 2000 in SSA countries. However, in 2016 this figure had increased to 48.136 people per sq. km. This continuous increase in population density leads to informal settlements (slums) and environmental pollution, and thus adversely affects life expectancy.

Contrary to the long-run results, in the short run, EPI and EV significantly reduced life expectancy in the sampled countries. This suggests that, in the short run, during the economic transformation from agricultural to industrial economies, manufacturing companies have limited waste management facilities or strategies and generally dump waste in rivers. Furthermore, in the short run, environmental quality in African nations is poor due to weak environmental protection strategies and awareness.

Nonetheless, urbanization positively significantly increases life expectancy in African nations, both in the short and long term. Government expenditures also makes a positive contribution to life expectancy in the short term. However, unemployment negatively reduces life expectancy of African people in the short run.

This is because, in the short run, unemployment can cause stress, arthritis, dementia, depression, hypertension, obesity, high cortisol and inflammation, poorer self-rated health, and suicide, and all these reduce life expectancy. In this context, several studies argue that unemployment leads to either mental or physical health problems (or both).^58–62^ Very recently, Medical Xpress^63^ noted that millions of people around the world have lost their jobs due to the COVID-19 pandemic and the resulting unemployment may lead to stress, anxiety, depression, and other mental health problems. Therefore, it is possible to conclude that in the short-run most Africans are not mentally prepared for job loss because most support their families. Furthermore, due to the absence of unemployment benefits and poor saving habits, in the immediate future unemployed people in Africa will suffer greatly, leading to stress, anxiety, depression, other mental health problems, and suicide. However, in the longer term, they can adapt to the situation or involve themselves in informal activities. Similarly, in the short run, per capita environment negatively and significantly affects life expectancy, which might be due to severe income inequality in African countries.

In both estimated models, the coefficients of one lagged period of the error correction term (ECM) were highly significant and fell between 0 and −1, which implies the existence of a long-run relationship among the variables. The coefficients of ECM are −0. 0423 and −0. 0410 for Models 1 and 2, respectively, which implies every year about 4% (too slow to be equilibrium) of the deviation from the long run is corrected.

### Robustness check analysis using alternative models

The present study conducted the Granger causality test in order to confirm whether environmental quality has a potential causality for life expectancy. The results demonstrated that the null hypothesis that change in both EPI and EV does not homogeneously cause LIFEXP is rejected at a 1% level of significance, suggesting that improved environmental quality can increase life expectancy *(Table 5).*

Since per capita GDP, which was used in the previous section, is comparable with either GDP growth (GDPGR) or GDP per capita growth (GDPPCG), this study estimated the alternative models to check the robustness of the results. That means both of the previous equations *(Equations 3 and 4)* are estimated by substituting GDPPCG by GDPPC *(Table 6).* The study also estimated the equations using the natural logarithm of life expectancy (LNLIFEXP) and GDPGR *(Table 7).* As an alternative result, the study used a natural log of life expectancy and GDPGR because all variables in the models were measured as either an index or percentage. Therefore, the study used the natural logarithm of life expectancy and GDPGR to consistently measure all variables and interpret the regression coefficients as elasticity (percentage).

Similar to the previous results, the robustness analysis also conducted a cross-sectional dependence test, panel unit root tests, and cointegration test before estimation. The result confirms that there is no cross-sectional dependence in any of the models and all alternative variables (LNLIFEXP, GDPPCG, and GDPGR) are stationary at level (I(0)). In addition, the Kao cointegration test confirms the long-run relationship among the variables in the model. To save space, this study does not report those results here, but they are available from the authors.

The long-run and short-run estimated results show that both EPI and EV have a positive and significant impact on life expectancy in African nations, which is consistent with the previous findings. The results in Table 6 confirm that a unit increase in EPI and EV increases life expectancy by 0.1134 and 0.137 years, respectively. Similarly, a unit increment in EPI and EV leads to a rise in life expectancy by 0.18 and 0.37%, respectively *(Table 7).*

## Conclusions

Protecting human health from risk is vital for human welfare, and social and economic development. Among the factors affecting human health, low environmental quality is a leading cause of death in LMIC countries. Currently, the SDGs have many targets for reducing environment-related deaths. Therefore, this study aimed to examine the impact of environmental quality on life expectancy in selected African countries between 2000 to 2016, employing the panel ARDL – DFE estimation technique. In long-run models, the study found that improvements in EPI and EV significantly increased life expectancy in African countries. The study recommends that African countries should give high priority to improving environmental quality by developing policies to stimulate environmentally friendly economies (green economies) using resources such as renewable energy (electricity generation, air and water heating/cooling system), effective land management (reforestation), and encouraging optimum use of water resources. In addition, the waste management systems of African nations should be improved. Furthermore, developing and applying strict rules and regulations to protect the environment and other technological measures can help in improving the quality of the environment as well as human health. Achieving rapid and sustainable economic growth (especially in productive sectors) is essential to provide basic health facilities, a better quality of life, and to increase longevity for the people of Africa. In addition, African states should work to improve the general level of education of their people, which would make it easier to raise awareness of the earth's natural resources. Africans also need demographic measures like a reduction in birth rates, which can substantially reduce population density and environmental pollution and improve life expectancy.

Finally, although the present study has tried to satisfactorily address the literature, measurement, and methodological aspects of the effect of environmental quality on life expectancy, it has limitations. Lack of data on environmental quality limits the time frame of this study to conditions after 2000, and therefore it did not take into account major policies and environmental agreements in the 1980s and 1990s such as health policy on primary health care, the United Nations framework on climate change, and the Kyoto protocol. In addition, due to data constraints on the main target variables (environmental quality) this study is limited to 24 African countries. Furthermore, it would have been interesting to estimate either one or two-step system GMM. However, the life expectancy (LIFEXP) variable is not integrated (I(0)) and all other variables are integrated at the first difference (I(1)) (*Table 3*); hence, this study could not employ the GMM estimation for an additional robustness check. Therefore, future studies should consider these factors in their analyses.
